# Data-Driven Approaches to Integrated Disaster Risk Management: Special Issue of the 9th Conference of the International Society for Integrated Disaster Risk Management

**DOI:** 10.1007/s13753-020-00324-4

**Published:** 2020-12-07

**Authors:** Vincent Lemiale, Mahesh Prakash, Ana Maria Cruz

**Affiliations:** 1 CSIRO Data61, Clayton South, VIC 3169 Australia; 2 Disaster Prevention Research Institute, Kyoto University, Kyoto, 611-0011 Japan

The 9th Conference of the International Society for Integrated Disaster Risk Management (IDRiM) was held in Sydney on 2–4 October 2018. The event was hosted by Data61, the data innovation hub of Australia’s National Science Agency CSIRO. The IDRiM annual conference series traditionally brings together researchers and practitioners across all disciplines of disaster risk management (DRM) and the Australian instalment was no exception. More than 120 participants from 20 countries attended the congress, representing all sectors of DRM including academia, industry, and governmental agencies, to exchange on ideas and best practices through a mix of plenaries, presentations, panel discussions, and poster sessions.

This special issue, entitled *Data*-*Driven Approaches to Integrated Disaster Risk Management*, mirrors the overarching theme of the 2018 IDRiM conference, which set out to explore the ever-increasing role of data in all areas of disaster risk and emergency management. The articles included in this special issue have been selected to represent the diversity of data-driven approaches under active development for DRM. They are also illustrative of the multidisciplinary nature of DRM, its applications ranging from natural hazards (such as earthquakes, tsunamis, hurricanes) to man-made disasters or even a combination of the two. All four phases of DRM, namely prevention (or mitigation), preparedness, response, and recovery are covered in this volume, and many of the themes that were extensively discussed during the conference are further explored here.

In the prevention phase, data science and analytics can inform us on the potential impact of both natural hazards and man-made disasters to help protect the ecological diversity of our environment and make better long-term choices and investments, which in turn will improve the safety and welfare of our communities, as discussed in Liu et al. In this context, the ability to access extensive databases is a critical element of building evidence-based strategies for prevention and preparedness purposes (Elwood et al.; Pinelli, Esteva, et al.). Important challenges remain, however, when dealing with heterogeneous sources of data and several of the technologies presented in the special issue aim to address the issues of data collection, data curation, and data sharing to dramatically improve such cross-disciplinary collaboration (Elwood et al.; Pinelli, Esteva, et al.; Luo et al.).

Digital technologies such as smartphones have become ubiquitous in modern life, but their application to DRM has yet to be fully realized. An example of such application is discussed in Yamori and Sugiyama, where a smartphone app was developed to increase the preparedness of the local community by simulating tsunami evacuation drills. Another example of the potential application of digital technologies is the development of evidence-based decision support systems both for planning (prevention, preparedness) and operational (response) purposes. As the volume and accuracy of available data increase, a number of initiatives around the world have been focussing on developing such systems in recent years to help emergency coordinators make informed decisions. Two of the articles in this special issue discuss these concepts as applied to storms, floods, and landslides in Japan (Takenouchi and Yamori) and debris flows in Indonesia (Hapsari et al.).

Improved resilience is achieved through risk assessment and risk reduction, which in turn implies understanding and reducing various sources of uncertainty in the data and in the processes at play. Two articles propose methodologies to address these important issues in the context of hurricane vulnerability (Pinelli, Cruz et al.) and post-earthquake lifeline systems recovery (Deelstra and Bristow). This last article in the volume also exemplifies that perhaps one area where the unrealized potential of data-driven approaches is the most evident is in fact in the post-disaster recovery phase. The premise here is to help communities regain a sense of stability and rebuild faster after being struck by a disaster.

Each of the articles in this special issue may be briefly summarized as follows:

*Variability in Regional Ecological Vulnerability: A Case Study of Sichuan Province, China* by Yimeng Liu, Saini Yang, Chuanliang Han, Wei Ni, Yuyao Zhu. The authors developed a data-driven method to assess the vulnerability of ecological systems to external stress factors such as natural hazards and human activities. They found that for the case study of Sichuan Province, anthropogenic construction activities increased ecological vulnerability more than any other factors considered, including natural hazards.

*Seismic Policy, Operations, and Research Uses for a Building Inventory in an Earthquake*-*Prone City* by Ken Elwood, Olga Filippova, Ilan Noy, Jacob Pastor Paz. A building inventory database integrating datasets from multiple sources was created for the city of Wellington, New Zealand to manage the risks associated with earthquake events. The multiple uses of such a database discussed in this article, ranging from planning to response, highlight the importance of easily accessible data in the decision-making process.

*Disaster Risk Management through the DesignSafe Cyberinfrastructure* by Jean-Paul Pinelli, Maria Esteva, Ellen M. Rathje, David Roueche, Scott J. Brandenberg, Gilberto Mosqueda, Jamie Padgett, Frederick Haan. This article presents a cloud-based cyber-infrastructure platform designed to address the challenges associated with collecting, curating, sharing, and analyzing heterogeneous datasets for disaster risk management purposes. The article discusses the application of this multi-disciplinary tool to several cases including soil liquefaction, seismic risk analysis, and hurricane recovery.

*Extracting Natech Reports from Large Databases: Development of a Semi*-*Intelligent Natech Identification Framework* by Xiaolong Luo, Ana Maria Cruz, Dimitrios Tzioutzios. This contribution explores the use of machine learning algorithms to extract information about natural hazard-triggered technological accidents from large databases for risk management purposes. It provides an example of using state-of-the-art computing technologies to realize the potential of available data for disaster risk management.

*Development and Social Implementation of Smartphone App Nige*-*Tore for Improving Tsunami Evacuation Drills: Synergistic Effects between Commitment and Contingency* by Katsuya Yamori and Takashi Sugiyama. Nige-Tore is a smartphone app that allows the user to participate in a tsunami evacuation drill. Participants take actions and choose escape routes based on real-time updates of a simulated tsunami displayed on a map. The authors discuss at length the benefits and challenges of the human-digital technology interactions brought about by this app, and explore how these new technologies may be useful resources to improve our preparedness for natural hazards and disasters.

*Synergistic Integration of Detailed Meteorological and Community Information for Evacuation from Weather*-*Related Disasters: Proposal of a “Disaster Response Switch”* by Kensuke Takenouchi and Katsuya Yamori. The importance of high-resolution meteorological data and weather forecasting models is discussed in the context of several common hazards in Japan, namely storms, floods, and landslides. From the results of this study it is apparent that the development of data-driven decision support systems is critically needed to assist local authorities develop appropriate and evidence-based emergency response, and to determine the timing of an evacuation.

*Naïve Bayes Classifier for Debris Flow Disaster Mitigation in Mount Merapi Volcanic Rivers, Indonesia, Using X*-*Band Polarimetric Radar* by Ratih Indri Hapsari, Bima Ahida Indaka Sugna, Dandung Novianto, Rosa Andrie Asmara, Satoru Oishi. A debris flow disaster warning system for a volcanic region in Indonesia is presented. The method estimates debris flow susceptibility through a simple data mining approach. The study also highlights the need for higher-resolution data to improve the robustness of the model.

*Uncertainty Reduction through Data Management in the Development, Validation, Calibration, and Operation of a Hurricane Vulnerability Model* by Jean-Paul Pinelli, Josemar Da Cruz, Kurtis Gurley, Andres Santiago Paleo-Torres, Mohammad Baradaranshoraka, Steven Cocke, Dongwook Shin. This contribution discusses the issue of epistemic uncertainty and approaches for uncertainty reduction with hurricane vulnerability as a case study, in the context of insurance claims. By integrating various sources of data augmented claims may be constructed, which then facilitate the development of a vulnerability model.

*Characterizing Uncertainty in City*-*Wide Disaster Recovery through Geospatial Multi*-*Lifeline Restoration Modeling of Earthquake Impact in the District of North Vancouver* by Andrew Deelstra and David Bristow. The authors studied, through a data-driven modeling approach, the recovery of lifeline systems in the earthquake-prone area of the district of North Vancouver. One of the uses of their model is to determine which systems are more likely to be restored first following a disaster and to allocate resources accordingly to accelerate the overall recovery process.

Following the 2018 IDRiM conference in Australia, the next congress was held in Nice, France, in October 2019 and focused on “knowledge-based disaster risk management” thus expanding on the notions of information and data science applied to risk reduction, resilience, and sustainable development. Two major events in 2019-2020 dramatically illustrated the relevance of these two themes of data- and knowledge-based approaches for disaster risk management.

The first was the catastrophic bushfire season that occurred across six states in Australia between the second part of 2019 and ended in May 2020. This event caused 34 direct fatalities and in excess of 400 indirect fatalities (through smoke inhalation) and affected almost three billion animals, not to mention the economic and broader ecological costs that are both yet to be fully estimated. Following this major disaster, it became clear that in order to become more resilient to such large-scale events as a society, better data analytics, artificial intelligence (AI) solutions, and new digital innovations were needed. The Australian government thus invested a further AUD 88.1 million over the next 10 years in the Bushfire and Natural Hazards Cooperative Research Centre, with space monitoring and predictions of natural hazards, development of new digital technologies, and AI solutions all at the heart of the center’s mission.

As catastrophic as the Australian bushfires had been, they were almost eclipsed in the media by the global COVID-19 pandemic that developed worldwide in 2020. Again, the role of data-driven science in the fight against the spread of the virus was abundantly clear. Many governments based their decisions to implement lockdown measures on predictive scientific models that utilized the latest available data to help assess the effectiveness of potential mitigation strategies in slowing down the spread of the virus. Moreover, geospatial monitoring has been used worldwide to manage this crisis, firstly through the development of smartphone apps to improve the enormous task of contact tracing, and secondly to understand the effect of restrictions on the mobility of individuals put in place by authorities.

Now more than ever, the world has therefore become acutely aware of the key role that data-driven approaches play in disaster risk management, and we hope that this special issue will provide the reader with an overview of some of the important initiatives currently pursued in this area.

Vincent Lemiale, Mahesh Prakash, and Ana Maria Cruz

Guest Editors

